# Dynamics of neurocognitive impairments in patients with chronic alcoholism of the second stage

**DOI:** 10.1192/j.eurpsy.2024.1298

**Published:** 2024-08-27

**Authors:** L. Baranskaya, E. Babyshkina, A. Sidenkova

**Affiliations:** ^1^Psychiatry, Psychotherapy and Narcology; ^2^Ural State Medical University, Yekaterinburg, Russian Federation

## Abstract

**Introduction:**

Neuropsychological disorders in patients with alcoholism intensively studied since the mid-70s of the last century. Research in this area divided into three groups: the study of premorbid neuropsychological features of alcohol dependence; study of neuropsychological disorders of chronic alcohol use; study of the prognostic value of neuropsychological disorders in patients suffering from alcohol dependence. In domestic neuropsychology, is the necessary information about the neuropsychological characteristics of patients suffering from alcohol dependence, neuropsychological manifestations in cognitive processes.

**Objectives:**

to identify neuropsychological features of patients suffering from alcohol dependence with a diagnosis of stage 2 alcoholic disease

**Methods:**

A neuropsychological examination was carried out according to the method of A.R. Luria of 39 patients aged 29 to 68 years with a diagnosis of stage 2 alcoholic disease. The group of patients is divided into 3 subgroups of alcohol abuse: up to 10 years, 10-20 years; more than 20 years.

**Results:**

Disorders of higher mental functions identified in all subgroups. In chronic alcoholic encephalopathy, there is a tendency to increase cognitive deficits. According to the results of the neuropsychological examination, it was found that the greatest disorders in patients of the first subgroup occur in the implementation of successive processes (memory, thinking), arbitrary regulation of activity, and also relate to the regulatory aspects of memory, attention, thinking and speech.

In patients of the second subgroup, the most numerous in this sample, violations of visual object gnosis were revealed, as well as a violation of the synthesis of information necessary to endow the image of the object with a certain meaning In patients of the third subgroup, pronounced disorders inherent in the first and second subgroups were found, as well as distortions in the identification of emotions, that is, the inability to compare emotional objects with an emotional standard, which indicates signs affective-cognitive deficit in alcoholic disease of the second stage.

**Conclusions:**

In the study, the dynamics of neuropsychological disorders in patients with alcohol disease of the second stage, depending on the experience of alcohol abuse, found

**Disclosure of Interest:**

None Declared

